# The Role of Public Nutrition Research Organizations in the Construction, Implementation and Evaluation of Evidence-Based Nutrition Policy: Two National Experiences in Mexico

**DOI:** 10.3390/nu11030594

**Published:** 2019-03-12

**Authors:** Juan A. Rivera Dommarco, Teresita González de Cosío, C. Gabriela García-Chávez, M. Arantxa Colchero

**Affiliations:** 1 Instituto Nacional de Salud Pública, Cuernavaca, Morelos 62100, México; 2 Centro de Investigación en Nutrición y Salud, Instituto Nacional de Salud Pública, Cuernavaca, Morelos 62100, México; teresita_1956@yahoo.com (T.G.d.C.); gabriela.garcia@insp.mx (C.G.G.-C.); 3 Centro de Investigación en Sistemas de Salud, Instituto Nacional de Salud Pública, Cuernavaca, Morelos 62100, México; acolchero@insp.mx

**Keywords:** malnutrition, program evaluation, nutrition policy, public health nutrition

## Abstract

Malnutrition and poor diet are the largest risk factors responsible for the global burden of disease. Therefore, ending all forms of malnutrition by 2030 is a global priority. To achieve this goal, a key element is to design and implement nutrition policies based on the best available scientific evidence. The demand for evidence-based nutrition policies may originate directly from policymakers or through social actors. In both cases, the role of research institutions is to generate relevant evidence for public policy. The two key objects of analysis for the design of an effective policy are the nutrition conditions of the population and the policies and programs available, including the identification of delivery platforms and competencies required by personnel in charge of the provision of services (social response). In addition, systematic literature reviews about risk factors of malnutrition, as well as the efficacy and effectiveness of policy actions, lead to evidence-based policy recommendations. Given the multifactorial nature of malnutrition, the drivers and risk factors operate in several sectors (food and agriculture, health, education, and social development) and may be immediate, underlying or basic causes. This multilevel complexity should be considered when developing nutrition policy. In this article, we show two models for the evidence-based design of nutrition policies and programs that may be useful to academia and decision makers demonstrated by two examples of policy design, implementation and evaluation in Mexico.

## 1. Introduction

Like many countries in Latin America, Mexico experiences a double burden of malnutrition, including undernutrition such as micronutrient deficiencies and stunting, in addition to overweight and obesity [1]. Both conditions are related to poor quality diets and imbalances in energy intake, while overweight and obesity are also associated with low physical activity [2]. Both diet quality and physical activity are determined by a wide range of factors at different levels of the social-ecological model. In 2005, globally, 35% of all deaths at any age and for both sexes were attributable dietary risks, 4.4% to low physical activity, 10.8% to high Body Mass Index (BMI) and 7.4% to child and maternal malnutrition (suboptimal breastfeeding, child undernutrition and iron, vitamin A and zinc deficiencies) [3]. However, 10 years later (in 2015), although the burden of child and maternal malnutrition had decreased to 4.4%, the burden of dietary risks, high BMI, and physical activity increased to 37.4%, 12.3%, and 5%, respectively. Thus, ending all forms of malnutrition by 2030 is a global priority.

Programs and policies in public health should be based on the best available scientific evidence, generated by research institutes with experts in the area of public nutrition [4,5,6]. It should be recognized that policymakers need to consider other criteria, in addition to scientific evidence, in the definition of policies that are likely to receive political support. However, often scientific evidence is ignored. This is due to several reasons such as lack of awareness of the evidence, poor understanding of the evidence, limited interaction and collaboration between policymakers and scientists when designing policies [7], political pressure, lack of consensus among policymakers, the power of other economic interests including the food industry that oppose certain policies and lack of mechanisms to declare and manage conflicts of interest [8].

This article presents two models that emerged from a retrospective analysis of two experiences in Mexico: (1) the design of the nutrition component of the conditional cash transfer program originally called *Progresa* implemented in 1997 and (2) the obesity prevention policies, particularly the implementation of fiscal policies in 2014. We chose these two experiences because they uniquely reflect how evidence was used for the approval, design and evaluation of the policies implemented in the country. These two experiences happened in very different political environments with interactions between diverse stakeholders. The first example was characterized by political will as a mean to achieve an objective, using evidence, open dialogue to interact with stakeholders and involve them in the design of the program, and under no resource constraint for implementation. In the second case, there was no consensus within the government (conflicting positions) so there was no easy ground for communication between those who generated the evidence and decision makers, therefore it was necessary to generate social support to achieve the goal.

## 2. Role of Public Nutrition Research Organizations in Contributing to Ending Malnutrition

**Figure 1:**
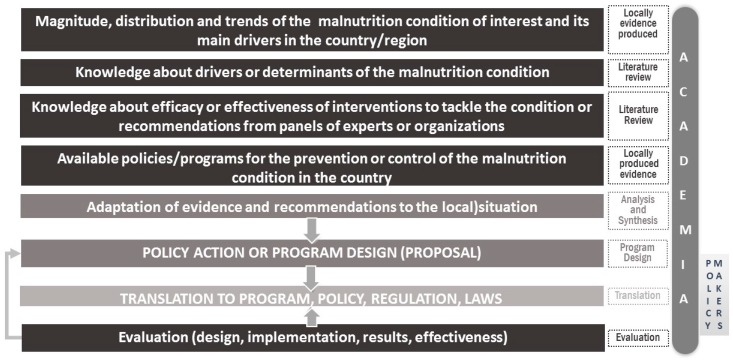
Model for evidence-based design of policies or programs Adapted from a published source [9].

The design of sound policy to tackle malnutrition in all its forms requires that research institutions develop strategic research agendas to bridge knowledge gaps. The process to design evidence-based policies or programs applied to malnutrition begins with a study of the nutritional conditions and main drivers in the country/region, which is locally produced evidence (Figure 1).

Research in public nutrition should consider two key objects of analysis. The first object is the study of the nutrition conditions where there are multiple factors beyond personal decisions that influence dietary choices such as culture, education, income, societal attitudes towards food, incentives, and motivation. Influences from the environment include the availability of food at school, local stores, supermarkets, and on the other hand, the food and beverage industry incentives, food safety, and food transportation and distribution, among others [10,11]. The study of the nutrition condition identifies the magnitude, distribution, and trends of malnutrition in all its forms in the population.

The second object is the study of the social organized response, which encompasses the policies and programs currently available, including the identification of delivery platforms and competencies of personnel for the provision of nutritional products and services, as well as health care and economic systems, international trade agreements, and other factors that influence the food supply and food environment [10].

The literature review will provide knowledge on the drivers or determinants of the malnutrition condition and the efficacy/effectiveness of interventions to tackle the condition. Also, recommendations from panels of experts or organizations can be used when available [9].

Systematic literature reviews about the risk factors of malnutrition in all its forms, as well as the effectiveness of an array of promising policy actions, are required for evidence-based policy recommendations. In other words, ongoing synthesis and evaluation are required. For some topics, international evidence-based recommendations developed by expert panels with clear criteria for identifying and classifying the evidence are already available. Examples of useful documents with expert science-based recommendations are available from the World Health Organization [12], the Pan-American Health Organization [13], the World Cancer Research Found International [14], and several Lancet Series [15] and commissions. 

Knowledge of locally produced evidence (i.e., magnitude distribution and trends of the nutrition conditions and policies and programs for their prevention or control) is essential for the analysis of the local conditions and social response and for the adaptation of the efficacious or effective intervention to the local conditions, which can then be translated into a program, policy, or law. This exercise will produce locally adapted evidence-based recommendations, which ideally should be tested in small-scale interventions for feasibility and efficacy before implementing them at a larger scale.

Given the multifactorial nature of malnutrition, the risk factors operate in multiple sectors (food and agriculture, health, education, and social development) and may be immediate risk factors (generally biological factors or individual behaviors), underlying (contextual factors that drive food intake, such as food environments), or basic causes (factors that occurs at a macro level and influences the underlying causes, such as technology, globalization, urbanization, and food processing). This multilevel complexity should be considered when developing policy recommendations for the prevention and control of malnutrition in all its forms. Likewise, for the adequate implementation of these policies, the government must have the will to act and work in an intersectoral manner with other government sectors, departments and ministries. Moreover, the support of civil society and non-governmental organizations is needed to support and monitor the implementation and sustain policies [10]. This complexity is a significant barrier to optimal interventions.

## 3. Demand for Nutrition Policy and Programs: Two Main Models Applied in Mexico

Who were the actors who identified the need for nutrition policy in Mexico? Through which mechanisms these actors identified this need? The demand for nutrition policy and programs in Mexico has originated through two different models. During the last decade of the 20th century, policymakers became interested in the use of evidence for the design of programs for poverty and undernutrition alleviation and sought the advice of investigators for that end. An example in Mexico was the interaction between investigators from the National Public Health Institute (INSP) with Mexican Government officials, for the design of the nutrition component of the conditional cash transfer program originally called *Progresa*, which was later renamed *Oportunidades* and *Prospera* at different points in time during the last 20 years. In this first model, the demand for evidence-based nutrition policy originated through the direct interaction between academia and policymakers who were interested in the design and implementation of evidence-based policy actions (Model I). 

In the second model, the demand came from the public, as a result of the interaction between academia, civil society, and the Government (Model II). In this model, civil society organizations, through mass media campaigns, were able to influence public opinion about the importance of public health and nutrition actions and generate public demand for such policies. Academic institutions and civil society organizations worked with those in charge of the program, policy, regulation, or law (legislative and executive branches of power) to design and implement policy actions. An example is the interaction between INSP and Civil Society Organizations, which eventually fostered the public demand for obesity prevention policies, particularly fiscal measures.

### 3.1. MODEL I. Interaction between the National Public Health Institute (INSP) and Decision Makers for Designing, Evaluating, and Improving the Nutrition Component of Progresa-Oportunidades-Prospera

**Figure 2:**
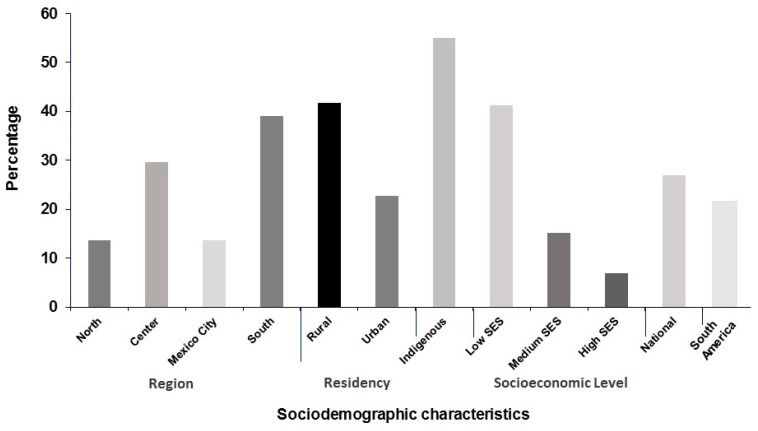
Prevalence of stunting in children <5 years in Mexico in 1988. Adapted from a published source [9], *N* = 7246 children <5 years, National Nutrition Survey 1988.

In the early 1990s, a research group at INSP analyzed a National Nutrition Survey conducted in 1988 and described the nutrition conditions of the country at the time. The results showed that Mexico had a much higher prevalence of stunting and anemia, given the size of its economy. One of the reasons for this high prevalence was the profound inequities among different social sectors in Mexico. For example, the prevalence of stunting in children <5 y of age (Figure 2) was 55% in indigenous households, 41.7% in rural areas, 41.2% in the lower socioeconomic tertile, and 39% in the southern region.

In contrast, the prevalence in the upper socioeconomic tertile was 7%, 13.6% in Mexico City, and 13.7% in the Northern states [16]. Moreover, the national prevalence of stunting in Mexico was 22.8% while the prevalence in the South American region as a whole was 17.2%. [17] Ironically, at that time, the Mexican government was investing large amounts of resources in food aid programs. In 1993 the Government invested almost 2 million dollars per day in food-aid programs, a much higher investment than estimates of funding required to tackle malnutrition.

At the time of these analyses, there was evidence in the literature about the larger effectiveness of programs that targeted nutrition interventions to low income and indigenous populations [18]. Furthermore, there was evidence of the beneficial effects achieved when targeting interventions in the first 1000 days of life, which constitutes a window of opportunity for the prevention of undernutrition [19].

#### 3.1.1. Why Were Investments in Nutrition Ineffective in Mexico?

**Figure 3:**
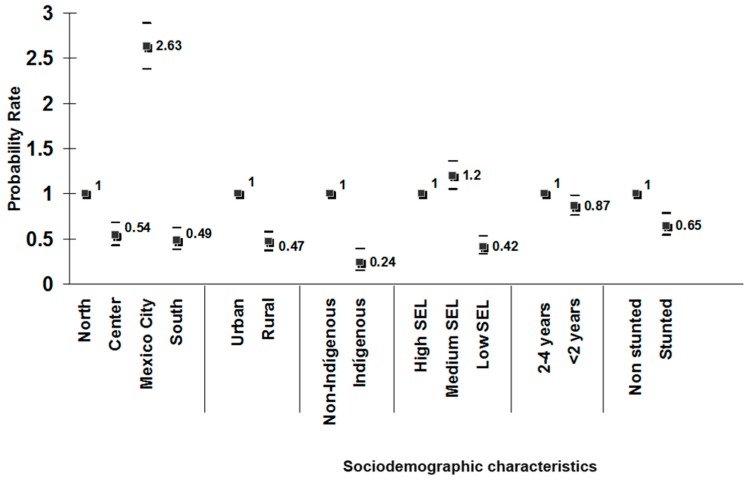
Probability rate (±95% C.I.) of participation in food programs in households with children <5 years of age in 1988. Adapted from a published source [9], *N* = 7246 children <5 years, National Nutrition Survey 1988.

INSP conducted a simple comparison between the distribution of stunted children <5 years by different regional and socioeconomic categories versus the proportion of families who were beneficiaries of food-aid programs in each of the subgroups studied. This straightforward exercise concluded that the food programs in Mexico were not targeted to those most in need [20]. For example, Figure 3 shows the probability rate of participation in food programs in households with children <5 years of age in 1988 by geographic region, urban and rural areas, indigenous and non-indigenous population, socioeconomic status, age category, and nutritional status (stunting) of children within the households.

The expectation under an adequate targeting scheme was that programs would be targeted to indigenous populations, rural areas, the southern region, those with low socioeconomic status, the younger age category (<24 months), and stunted children. However, the results showed that in all cases, the probability of receiving the benefits of nutrition programs was lower in the vulnerable categories, where the need for nutritional support was greatest. It was therefore concluded that despite the high investment of the Mexican Government in food and nutrition programs, these were not targeted to the right subpopulations and therefore, did not achieve intended results.

#### 3.1.2. Summary of Reasons for Low Effectiveness of Food Programs in Mexico

In addition to the inadequate targeting of the food aid programs mentioned before, other reasons for the food program’s lack of effectiveness included the nutrition content of the foods distributed to families in these programs. Foods distributed included *tortillas*, the Mexican staple which is made from lime-treated corn or cooking oil. These and other foods distributed were poor sources of key micronutrients that were essential, given the existing levels of stunting, anemia, infection and arrested cognitive development associated with poor diets among infants and young children. Without these micronutrients, particularly zinc [21] and iron [22], the growth and development of children would be affected. Moreover, the foods distributed were not necessarily part of the dietary traditions of young children in underserved communities. Furthermore, investment in communication and education strategies was weak or inexistent.

Although there was a large variety of food aid programs, their components or benefits were often duplicated and their actions uncoordinated. All these flaws were often inadvertent because the programs’ impact was not evaluated, so there was not an opportunity to gauge their benefits nor the convenience of modifying or eliminating them. 

As a result of all the reasons presented above, the malnutrition prevalence remained high, and the decline in the prevalence of stunting, an indicator of chronic malnutrition, was less than what was expected during the 1990s, given the high investment in food-aid programs [9,23].

There was a clear need for evidence-based information to lead program and policy design and to identify causes of the low efficacy or effectiveness of nutrition programs, but this need was not clear to most policymakers.

#### 3.1.3. Interaction between INSP and the Mexican Government for the Design of the Nutrition Component of PROGRESA

Given the results of this analysis, researchers from INSP issued recommendations based on adequate targeting. This included focusing nutrition interventions to low income and indigenous households, rural areas, pregnant women, and children <2 years, as well as utilizing evidence-based interventions, such as nutrition education, complementary foods specifically designed for children <2 years, *coordination of programs and actions* to improve efficiency, and finally, monitoring and evaluation embedded in the program and design.

##### Design of Progresa Based on the Evidence Presented and Recommendations

In 1997, *Progresa* was created and a nutrition component was included. The program included conditional cash transfers used as incentives for investments in nutrition, health, and education. Women received the transfers that were conditional on compliance with health, nutrition, and school services. The program also provided fortified food supplements for children and women and included a nutrition education component targeted to low-income households, rural areas (during first phases), households with children <2 years of age, and pregnant and lactating women. A key component included from the beginning of the program was a robust evaluation [23,24]

##### Fortified Food Supplement Composition

One of the recommendations was to provide micronutrient-fortified foods for women and children. The food supplements were targeted to the groups of individuals that are more likely to benefit from the product: children aged 4 to 23 months, underweight children aged 2 to 4 years, and pregnant and lactating women in the low-income beneficiary households. The supplements were designed by a group of experts in nutrition, according to evidence on the nutritional status of the population for whom the supplements were intended. The main ingredients were dry whole milk, sugar, maltodextrin, and the micronutrients most deficient in their diet. The supplements were distributed at health centers. Mothers were instructed to add four spoons of boiled water to one ration, which produced a puree consistency product, referred to in Spanish as papilla called “Nutrisano” [23,25].

**Figure 4:**
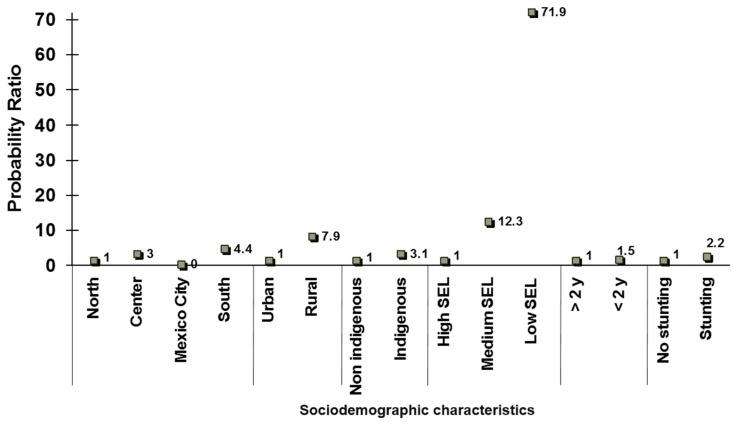
Probability rate (±95% C.I.) of participation in Food programs in households with children <5 years of age in 1999. Adapted from a published source [9], *N* = 8011 children <5 years, National Nutrition Survey 1999, Stunting or no stunting in the household.

An immediate effect of *Progresa* was the reversal of inadequate targeting. As shown in Figure 4, in 1999, after the implementation of *Progresa*, the probability of receiving the benefits of nutrition programs were higher in all categories in need of nutrition interventions. It was therefore concluded that Progresa was indeed targeted to the right subpopulations [9].

##### Socio-Political Drivers

The implementation of *Progresa* was characterized by political will that enabled a successful interaction between INSP researchers and the government. Key decision makers at the Ministries of Finance, Health and Social Development at the Federal Government were supportive of using the evidence for the design, implementation, and evaluation of *Progresa* because they were experts in the field and/or had prior academic training. In addition to political will, the government had enough resources and interest in implementing programs to reduce undernutrition. The initiative had international support as most of the recommendations were validated by international agencies such as the World Bank that shared similar goals of designing programs to reduce the intergenerational transmission of poverty.

### 3.2. MODEL II. Interaction between INSP-Civil Society Organizations to Foster the Demand for Obesity Prevention Policies

The battle against obesity in Mexico has involved the participation and interaction of three players: research institutions, lobbyists and advocacy organizations, and legislative and executive branches of Government. Working together, these institutions and organizations were able to shape one of the most effective policy actions for the prevention of obesity, that is, the approval and implementation of fiscal policies as disincentives for the consumption of unhealthy beverages and foods [26]. From 1988 to 2012, the prevalence of obesity in Mexican women increased from 9.5 to 24.9% and this upward trend was sustained until 2006 (from 24.9% to 32.4%); while the prevalence of overweight increased between 1988 (25%) and 1999 (36.1%) but remained stable from 1999 to 2006 (36.9%) [27].

### 3.3. Risk Factors in Mexico

The persistence of undernutrition and obesity in low and middle-income countries, such as Mexico, are related to both poor quality diets and positive energy expenditure [2]. Analysis from the National Health and Nutrition Surveys 2012 characterized the food consumption of the Mexican population and found a high contribution to total energy intake of sugar-sweetened beverages (SSBs) and high energy-dense nutrient-poor foods [28,29,30,31]. Reports of expert consultation from World Health Organization (WHO)/Food and Agriculture Organization (FAO) [32,33] and World Cancer Research Fund International (WCRF)/American Institute for Cancer Research (AICR) [14] have identified several risk factors for obesity, including sedentary living, consumption of SSBs, high energy-dense foods and fast food, and time spent viewing television. On the other hand, an increase in physical activity, being breastfed, and low energy-dense food intake may lead to decreased risk (protective factors) [14,33,34].

#### 3.3.1. Dietary Risks

A group of researchers from INSP provided evidence about a positive association between dietary energy density (DED) and excess body weight in school-age children. The mean DED was 175 kcal/100 g; only 2.5% of the study population consumed ≤125 kcal/100 [35], which is the WCRF dietary density recommendation to avoid obesity [14].

In addition, INSP found that in Mexican adults, basic foods groups (considered essential for a healthy diet) contribute 74% of total energy intake, while the remaining 26% is accounted for by discretionary foods, which are not essential for a healthy diet, are high in sugar and/or saturated fats, and are mainly SSBs and high energy-dense nutrient-poor foods (ultra-processed foods) [28,30].

##### Dietary Goals for Mexico Based on the Contribution of Food Groups to Total Energy Intake in Adults

**Figure 5:**
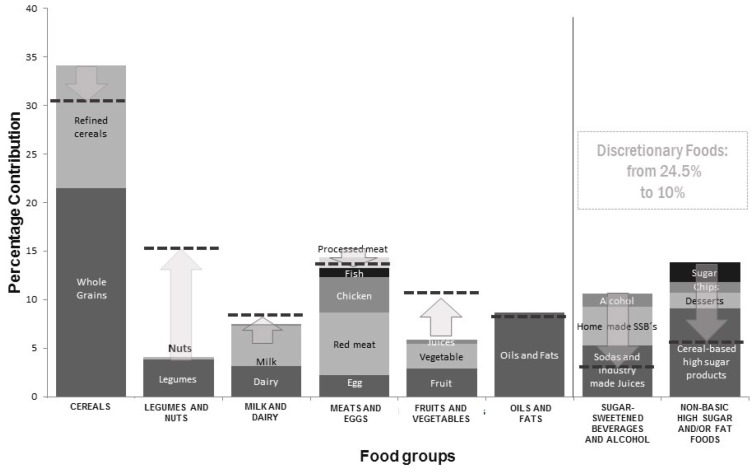
Dietary goals for Mexico based on the contribution of food groups to total energy intake (adults).

Due to the high-energy consumption derived from low quality food, establishing dietary goals in the Mexican population can help decision makers plan strategies for the prevention of obesity and other chronic diseases. Figure 5 shows dietary goals established for Mexican adults where the dotted lines mark the goal of consumption to be reached of each food group.

Dietary goals for Mexican adults involved substantially increasing the consumption of fruit and vegetables, more than double the consumption of beans and other legumes, and in a smaller proportion increasing the intake of milk and dairy products. On the other hand, consumption of SSBs, high energy-dense nutrient-poor foods, and alcohol should be greatly reduced from the current 24.5% of total energy to 10% or less. In addition, consumption of processed meat should also be minimized and to a lesser extent, red meat and refined cereals [36].

#### 3.3.2. Dietary Intake of Added Sugars (AS) in the Mexican Population

In Mexico, the mean consumption of added sugar from total energy is 12.5%, which is equivalent to 60 g/d [37]. WHO recommends consuming no more than 10% of the total energy intake, but preferably, less than 5%, which is equivalent to 25 g/d. Around two-thirds of Mexicans (57%–85%, considering each age group) are consuming quantities of over 10% of the total energy from added sugars [38]. SSBs constitute about 70% of all added sugars in the Mexican population [37]. The consumption of SSBs starts early in life. For example using 24 h recall methodology, 12% of infants < 6 months consumed non-dairy SSBs, 36% of children 6–11 months and 65% of children 12–23 months consumed this product on the day prior to the dietary survey [31]. Given the strong evidence about the deleterious effects of SSBs and added sugar [39,40,41,42,43], reducing the consumption of sugary drinks was one of the country’s top health priorities in Mexico.

### 3.4. Taxing Unhealthy Food as One of the Components of a Toolkit for Obesity Prevention

In 2012, the prevalence of overweight and obesity in Mexico reached 70% among adults and 30% among children [44,45] Although obesity and related diseases are the result of multiple causes, several systematic reviews and meta-analyses have shown positive associations between the consumption of SSBs and weight gain, diabetes, and other chronic diseases [39,40,41,42,43].

Fiscal policies have been used as a strategy to reduce the consumption of goods that are associated with negative effects on health such as tobacco and alcohol [46,47]. These taxes aim to correct for externalities associated with three market failures [48]: incomplete information on the effect on health of consuming these goods; externalities associated with direct and indirect costs of the diseases associated with the consumption of harmful goods; and, preferences that make individuals place more value on the immediate satisfaction of consumption over future health consequences.

In January 2014, Mexico implemented an excise tax of one peso per liter (approximately a 10% increase in price) on all non-alcoholic beverages with added sugars, excluding beverages with artificial sweeteners and 100% juices. The tax is paid by the producer but reflected in prices paid by consumers. The tax is adjusted when the cumulative inflation reaches 10% since implementation. A positive aspect of the tax is that it is higher for larger package sizes that are relatively less expensive (in pesos per liter) compared to smaller packages.

#### 3.4.1. Socio-Political Drivers and Synopsis of Process that Led to the Approval of the Tax

The implementation of the tax faced a very different political environment compared to Model 1 (*Progresa*). As expected, strong opposition of the tax came from the beverage industry throughout the full process: before, during, and after the tax was implemented. The strong power of the industry resulted in political pressures that fragmented the support from the government. Although the Ministry of Finance and the executive branch of the government supported the tax and took advantage of tax reform to introduce the SSB tax (partly due to the need for additional revenues), other key sectors, including Health, were not involved in the process. In this political context, the success of the implementation and continuation of the tax was a combination of actions from different stakeholders.

Due to the interaction among research institutions, lobbying and advocacy organizations, and legislative and executive branches of Government, a champion senator presented the first proposal for a 20% tax to SSBs in 2012 [49]. In 2013, the executive branch of government proposed a one peso per liter SSB tax that was approved. The Senate recommended the use of the revenues for obesity prevention and provision of water in schools.

Despite the strong opposition of the powerful Mexican beverage industry, the approval of the tax was the result of a combination of actions, evidence gathered, and favorable timing: an incoming administration and Congress with a need to raise revenues; an intense public debate in diverse forums on obesity; the food environment and the high intake of SSBs in the country; and two essential elements: a strategic partnership between civil society and academia, and strong scientific evidence.

##### Strategic Partnership

Three groups were instrumental in the design and approval of the tax. The first is an advocacy group “Alianza por la Salud Alimentaria” that combined a large group of civil society organizations led by “El Poder del Consumidor”. They used different strategies to raise awareness about the negative effects associated with SSB consumption through media campaigns and social media.

The second is a lobbying organization led by ContraPESO that worked on raising awareness among members of Congress and identifying key political moments for action, and champions to lead discussions towards the approval or continuation of the tax.

The third actor was the INSP that provided the evidence used for the design of the tax. INSP analyzed and compiled key scientific evidence that was used for the design of the tax. INSP had estimated through the National Health and Nutrition Surveys (1999, 2006, 2012) the magnitude, distributions (by age, gender, socioeconomic status and regions), and the trends of overweight, and obesity in Mexico over time. INSP also estimated the own and cross-price elasticities of demand for SSBs, which showed that the demand for these products was elastic and higher among low-income households and that the substitutes were water and other healthy beverages [50]. Price elasticities served as an estimate of the potential impact on consumption after a tax was implemented and were also used to estimate potential revenues. Potential impacts on consumption were estimated for the general population and by income groups, INSP also summarized the evidence through systematic literature reviews on the effects of SSB intake on obesity, diabetes, and other chronic diseases, and identified the potential uses of revenues for obesity prevention.

The approval of the tax is a public health success story, which resulted from the generation of evidence around a risk factor (SSBs) and the proposal of fiscal policy action to discourage the intake of this product and the use of that evidence by civil society and the Government.

## 4. Role of Evaluation

Evaluation is essential to provide feedback to policymakers. This should include assessing the design, implementation, and effectiveness. Design evaluation is inexpensive and is not time-consuming, making it a cost-effective tool to avoid the implementation of flawed policies or programs. Implementation of evaluation is essential for the success of a well-designed policy and provides timely feedback for improvement. Effectiveness evaluations assess the results of programs and policies on behaviors (i.e., food purchases, healthy eating, and physical activity) and nutrition outcomes (i.e., nutritional status). The results of the evaluation guide decisions about keeping or modifying policies or lead to recommendations for improving their design or implementation. They may also generate new research questions that feed the research-to-policy cycle. The whole process for the generation and evaluation of policies should include provisions for avoiding conflicts of interest.

An example of the evaluation of the two policy actions presented to illustrate each policy demand model are described in the next sections.

### 4.1. Evaluation of Progresa-Oportunidades-Prospera Program

Due to logistic and economic reasons, the initial stages of *Progresa* did not reach the total target population; therefore, the study design to evaluate the program was planned, taking advantage of the implementation of the program in phases, with a randomized design that included only 300,000 households, a small fraction of the target (6 million households). The evaluation began in 1998 in localities that were to be randomized to a group that would receive the program benefits and would be followed during two years or a group that would not receive the benefits immediately but would receive them immediately after two years when the study for the evaluation would be concluded. This second group would act as a control group. However, in the period of 1999–2000, due to political pressure, the control group also received the benefits of the program. Therefore, the group that received benefits of the program from the beginning was deemed the intervention group, and the second group, which received the benefits starting at the second year, was referred to as the “crossover intervention group”. Regardless of this study design modification, it was possible to evaluate the effect of the program over two years.

It was expected that the greatest effects would be observed in children who were randomized to the intervention group (who received the benefits of the program during the critical two first years of life). In contrast, the effects on infants in the crossover intervention group, who received the benefits of the program only during the second year (for a shorter time and during a less critical period), were expected to be lower. It was also expected that the greatest effect would be found in children from the lowest income level. Despite the fact that all households in the *Progresa* program belonged to the lower socioeconomic tertile, those in the lower half of this group had a substantially higher prevalence of stunting. For these reasons, all comparisons were made after groups were stratified by age (<6 months and 6–12 months at enrollment) and socioeconomic level (lower than average or higher than average).

The results of the evaluation showed that, as expected, the greatest effect on growth was observed in infants younger than six months and from the lowest socioeconomic level. The effect on linear growth in this group was 1.1 cm higher compared to the group who received the benefits only the second year. In addition, the positive effects of the program were observed in lower rates of anemia and higher food purchases. Finally, dietary effects on children were documented to be due to the intake of the nutrition supplement “Nutrisano” [51]. No effect was found in the group of infants from higher socioeconomic levels, nor in the group of older children [9,23].

#### Evidence from the Evaluation of the Nutrition Component of Progresa Used for Improving Its Design and Operations

Results from this evaluation allowed the identification of strengths of the program and areas for improvement. The positive effect of the program on linear growth and reduction of anemia deemed the program effective.

The evaluation generated new research questions and further studies to identify opportunities for improving the impact of the program. One of the studies concluded that the education component of the program was of low quality. As a result, a communication strategy was designed using state of the art methods and was tested at a small scale. Results of small scale testing showed that the program improved several behaviors aimed at increasing the intake of the supplements [52]. 

In addition, a follow-up study was carried out after finding that the reduction of anemia and iron deficiency was lower than expected. Further evaluation showed that the type of iron used had low bioavailability, so a reformulation of the supplements was necessary using forms of iron that proved to be bioavailable [9,53]. Currently, the program uses the forms of iron recommended by the study.

Another finding was that dietary effects were restricted to the intake of Nutrisano, not to improvements in overall infant feeding practices; this led to including the promotion of breastfeeding in the communication strategy and appropriate complementary feeding.

In summary, the evaluation of the program not only documented the positive effects, but it also resulted in other studies that led to changes in the design and evaluation of the program and thus, greater impact in the population.

### 4.2. Tax Evaluation

Several papers have been published documenting how prices, household purchases, and employment changed after the SSB tax was implemented in Mexico.

In urban areas, the tax passed along to the prices paid by consumers for all SSBs, i.e., prices increase on average by the amount of the tax (1.08 pesos per liter) [54]. Results from rural areas showed that the SSB tax did not pass completely through prices: prices increased on average 0.73 pesos per liter as opposed to the full peso in urban areas [55]. 

Several studies evaluated changes in taxed and untaxed beverages. A first study showed a 6% average reduction in taxed beverages in 2014 compared to predicted purchases based on pretax trends (2012–2013) in a panel of households living in 53 cities with more than 50,000 inhabitants [56]. A second study analyzed changes in purchases in the second year of implementation [57]. This study revealed that a reduction in taxed beverages was larger in the second year (9.7% decline). The studies revealed that reductions were observed in all socioeconomic groups but were more pronounced for the lowest socioeconomic groups [56,57,58], among residents living in urban areas, and households with children [58]. All studies show increases in untaxed beverages, particularly bottled water [56,57,58].

A recent study showed that employment in the beverage industry and at commercial stores did not decrease after the tax was implemented [59].

In summary, studies have shown that the SSB tax was effective at increasing prices and reducing consumption. The policy did not affect employment and potential effects on health, in the long run, have been estimated in simulation models [60,61].

It is important to note that taxes are only one component of a toolkit for obesity prevention. Fiscal policies should be combined with other strategies to reduce the burden of non-communicable diseases such as regulation of the availability of food and beverages in schools, implementation of consumer-friendly front of pack labeling, regulation of food marketing to children, and promotion of information on diet and physical activity for public awareness.

## 5. Conclusions

The design of policy actions for the prevention of malnutrition in all its forms requires scientific evidence. Research institutions in the field of public nutrition are essential to generate this evidence. These organizations should develop research agendas aimed at developing evidence for action. The examples presented in this paper for Mexico show that the political context can enable or prevent a favorable interaction between academia and decision makers. When there is no consensus within the government or political pressure, other stakeholders can work in strategic partnership to convince and push public policy.

The translation of scientific evidence to policy actions requires direct interaction between research institutions and policymakers (Model I) or civil society in order to generate public demand for policy action (Model II). In both cases, international and local evidence must be used to design evidence-based policy actions adapted to the local situation. Ideally, evidence-based locally relevant policy actions should be tested in small-scale interventions for feasibility and efficacy before implementing at scale.

In some instances, when immediate action is required, locally adapted recommendations are implemented without small-scale testing. In all cases, policies or large-scale programs should be evaluated, so that the body of evidence about effective policy actions is produced. In any case, the evaluation of policy or programs is key to provide feedback to policymakers.

The experiences described in this paper can be used as models in other countries under two scenarios: a political context enabling the interaction between decision-makers and academia in the use of evidence to design and implement a policy, or, in contrast, a context that requires the involvement of other stakeholders to generate social support.
